# Adolescent Vulnerability to Internet Media Exposure: The Role of Self-Mastery in Mitigating Post-Traumatic Symptoms

**DOI:** 10.3390/ijerph22040589

**Published:** 2025-04-09

**Authors:** Michelle Slone, Ayelet Peer, Michael Egozi

**Affiliations:** 1 Baruch Ivcher School of Psychology, Reichman University, Herzliya 4610101, Israel; 2 School of Psychological Sciences, Tel Aviv University, Tel Aviv 6997801, Israel; ayeletgilady@tauex.tau.ac.il (A.P.);

**Keywords:** internet media exposure, armed conflict, adolescence, post-trauma symptoms, self-mastery, resilience

## Abstract

The internet has revolutionized communication, becoming central to daily life. Consequently, news consumption shifted dramatically with increased media access, exposing individuals to global traumatic events, such as armed conflicts. Adolescents are particularly vulnerable to the negative effects of this exposure due to their media expertise and developmental stage. Young adults are more mature and independent but remain vulnerable to the harmful effects of internet exposure. This study examined the relationship between internet media exposure to armed conflict and post-traumatic symptoms and psychiatric symptomology among adolescents and young adults. Additionally, self-mastery was explored as a resilience factor in both groups. A sample of 329 participants, including 159 adolescents (ages 12–18) and 168 young adults (ages 20–26), completed questionnaires assessing direct and internet media exposure to armed conflict events, self-mastery, post-traumatic symptoms and psychiatric symptomology. Structural equation modeling (SEM) revealed that internet media exposure was positively associated with post-traumatic symptoms and psychiatric symptomatology only among adolescents, whereas direct exposure was significantly related to post-traumatic symptoms only among young adults. Self-mastery moderated these effects in both groups, buffering the psychological impact of the most relevant exposure. The findings underscore the need for interventions that foster self-mastery to mitigate the adverse effects of traumatic media exposure, particularly among adolescents. Developmental implications are discussed.

## 1. Introduction

The internet emerged as a groundbreaking innovation in communication and has grown into an essential part of daily life, offering many opportunities for information sharing, social interaction, entertainment, and more [1]. Social media platforms, which form a dominant aspect of internet use, are designed to encourage prolonged engagement that draw users into cycles of endless scrolling and result in hours of time spent on these platforms [2]. Alongside this shift in communication patterns, consumption of news has also changed dramatically, with many turning to digital and social media sources for updates. As smartphones, tablets, and computers make media more accessible than ever, individuals are exposed to greater amounts of information, including news about proximate and distant collective traumatic events such as armed conflict events and natural disasters. These media-driven exposures have been linked to emotional distress, particularly when they present graphic and disturbing images of violence in real time [3,4]. The growing frequency of such events, coupled with their widespread media coverage, amplifies the psychological impact on individuals, especially adolescents and young adults. The present study aims to examine the psychological effects of internet media exposure to armed conflict events among adolescents and young adults and the potential role of self-mastery as a moderator of this relation.

### 1.1. The Rise of the Internet and Social Media

The rise of social media has brought both opportunities and challenges. While these platforms enable unprecedented connectivity, they also present risks to well-being. Prolonged social media usage has been linked to a wide variety of psychological distress indicators, including psychiatric symptoms, sleep disturbances, and body image concerns [5]. A review of 43 empirical studies found that excessive social media engagement is associated with decreased participation in real-life social communities, lower academic performance, and strained interpersonal relationships, all of which may signal potential addictive behaviors [6].

Over the years, internet usage has grown steadily [7,8], with many turning to online news sources and social media for updates, a trend that has been progressively rising [9]. These changes have also transformed the ways in which news is accessed and consumed, shifting the focus from traditional media to digital and social platforms. Accordingly, nearly two-thirds of Americans reported obtaining news via social media platforms such as Facebook and Twitter. Around half of them access news from television. In contrast, radio and print have become less common news sources, with only 25% and 18% of Americans, respectively, using them regularly [9].

The likelihood of encountering a collective traumatic event, defined as a shared traumatic experience affecting large groups of people, such as armed conflict events or natural disasters, has grown significantly due to the omnipresence of modern media. Although such disasters are relatively rare, the 24 h news cycle ensures that these events are broadcast widely, reaching individuals far removed from the event itself. This constant exposure amplifies the potential impact of collective trauma, making it critical to recognize ways in which the media serves as a powerful conduit for shared distress [10].

### 1.2. Internet Media Exposure to Armed Conflict Events

The growing incidence of war, armed conflict, and terrorism highlights the global nature of these challenges that deeply impact societies and civilians, triggering widespread anxiety, fear, and feelings of helplessness [11,12,13]. Reliance on digital platforms and social media for news during such collective trauma events can amplify emotional distress. Users are often exposed to graphic, immediate, and frequently horrifying scenes of violence and destruction, which are shared in real time, precipitating anxiety and confusion. In addition to these unsettling images, individuals are frequently confronted with unverified rumors and conflicting information, further intensifying the psychological impact [3].

Media platforms such as Facebook, Twitter, Instagram, and TikTok have been widely adopted not only by general users but also by military organizations and terrorist groups. For these groups, the internet offers unique opportunities to extend their reach and influence and to maximize the dissemination of propaganda material [14]. Media coverage has the power to captivate audiences, evoke emotions, and dramatize events [15,16], often magnifying factual content and heightening perceived threats [15,17,18]. The significant advantage of these platforms is the ability to publish content freely without the need for oversight or approval from authorities [19,20,21,22]. The digital nature of internet content allows it to be endlessly copied and shared, making it a potent tool for radical groups aiming to broaden their audience globally. This is especially relevant in conflict zones where exposure to armed conflict scenes and terrorist-related media content has become an unavoidable part of daily life [23]. For many, smartphones and computers act as portals to violent and disturbing content, especially for younger users. Graphic depictions of armed conflict can be accessed instantly, creating a visceral and immediate experience of global events that were once perceived as distant [10,20].

Indirect media exposure to traumatic events such as armed conflict events is not classified as a Criterion A traumatic event in the DSM-5 Post-Traumatic Stress Disorder diagnostic criteria. However, individuals who are indirectly exposed to trauma through the media may still perceive these events as significant traumatic experiences [10]. A large body of research has highlighted the strong link between media exposure to collective traumas, particularly armed conflict, violence and natural disasters, and subsequent physical and mental health outcomes [10]. Countless individuals experience this indirect exposure, which can lead to profound psychological effects [19,24,25,26], including post-traumatic stress symptoms, anxiety [27,28,29], general distress and other psychiatric symptoms [30], heightened perceptions of threat and an increased likelihood of self-protective and avoidant behaviors [31]. Exposure to armed conflict events through the internet and social media was shown to have a significant negative impact on individuals of all ages. However, the heightened vulnerability associated with the sensitive period of adolescence may place adolescents at even greater risk for the psychological effects of such exposure. In light of this, the present study investigated potential differences between the responses of adolescents and young adults to this exposure, aiming to better understand the specific harmful effects experienced by each age group. Furthermore, the study explored the potential role of self-mastery as a protective factor that may moderate psychological effects for both adolescents and young adults.

### 1.3. Internet Media Exposure to Armed Conflict Events Among Adolescents

Social media has become an integral part of adolescent life, with studies indicating that nearly 97% of adolescents are active users today, spending an average of seven and a half hours each day on screen media. Additionally, 46% of adolescents report being “almost constantly” online [32,33]. Research on the emotional effects of social media use has identified impacts on self-esteem, social connection, internalizing issues, risk-taking behaviors, and sexual self-exploration [34,35,36,37,38]. As a result, understanding the impact of social media on adolescent well-being has become a universal concern [39]. This is also particularly relevant in the context of adolescents’ exposure to media coverage of war and armed conflict, as new technological platforms facilitate the rapid online distribution of graphic and disturbing content related to these events, accessible to anyone around the world.

Adolescents are especially susceptible to the psychological impact of exposure to violent media content due to both the developmental characteristics of this turbulent period [40,41] and adolescents’ high proficiency in using new media platforms. The developmental stage of adolescence is marked by a striving for autonomy, exploration, and adventure, often leading to engagement in risk-taking behaviors. This, combined with adolescents’ adeptness at navigating new media platforms and their easy access to violent content, produces greater susceptibility to the harmful effects of disturbing stimuli, especially given their relative immaturity in coping with such material [42,43]. In conflict zones, the prevalence of violent media content makes it nearly impossible for adolescents to avoid exposure, leading to continuous and frequent consumption of distressing material [25,44].

The psychological consequences of repeated exposure can vary, including heightened anxiety and general distress [26], aggressive behavior [44,45], and post-traumatic stress (PTS) symptoms [44]. These findings underscore the critical need to understand the unique vulnerabilities facing adolescents in relation to internet media exposure to armed conflict events and to explore potential moderating factors that can mitigate such harmful effects. These findings emphasize the critical role of parents in reducing the potential risks associated with adolescents’ internet use. Research has shown that parental monitoring of online activity helps mitigate the negative effects of media exposure [46,47], with effective monitoring serving as a protective factor against adverse online experiences, particularly in the context of social media [47]. While restrictive strategies, such as setting time limits or content controls, can contribute to safer internet use, research suggests that parental communication strategies that promote adolescents’ understanding and critical thinking about online content are even more effective. Such active monitoring approaches are associated with better adolescent self-regulation in media use [48,49].

### 1.4. Internet Media Exposure to Armed Conflict Events Among Young Adults

Emerging adulthood is a distinct developmental period during which individuals navigate a complex transition from childhood dependency to adult independence. It is characterized by identity exploration, self-focus, feeling “in-between”, and a sense of possibilities [50,51]. This stage involves significant life changes, such as leaving home, entering the workforce, or starting a family, all of which are integral to social, cognitive, and psychological development [50]. As emerging adults lose the structured support provided by schools, families, and social services, they must rely more on their own resources to navigate an unstructured environment [52]. While this period fosters identity formation and emotional growth, it also exposes young adults to increasingly complex and stress-inducing social environments, which may intensify the challenges they face [53].

Despite the close age proximity between adolescents and young adults, there are substantial differences in brain development, even after accounting for various physiological, psychological, and social factors [53,54]. Brain maturation continues well beyond adolescence, extending into the third decade of life, with key developmental changes in mentalization, theory of mind, and executive functions [53,55]. These advances in cognitive abilities may explain the more nuanced understanding of the social world among young adults and their higher capacity for self-awareness and attribution of complex mental states to others in comparison to adolescents. The accumulation of new social experiences and the ability to navigate social contexts becomes more sophisticated, aided by these cognitive developments [56].

Nearly all young adults today report having access to a smartphone, with about 20% identifying as “dependent” on their devices [33]. While the detrimental effects of excessive internet and social media use are well-documented among adolescents, these concerns extend into young adulthood [57], though the experience is often shaped by the different psychological and social stages of this life period. Media exposure to armed conflict events was shown to significantly impact the emotional well-being of young adults, predicting a broad spectrum of adverse outcomes. Studies have linked such exposure to post-traumatic stress symptoms [28,29,58] and, in some cases, full-blown PTSD [28]. Chronic stress symptoms have also been documented in response to media exposure years after a traumatic event [58]. Psychological distress is a common response [59,60], with evidence suggesting associations between media exposure and heightened levels of general sleep disturbances, including nightmares and nocturnal arousal periods [60]. Furthermore, increased social media use has been correlated with emotional distress, anxiety, depression, and somatic symptoms [59], emphasizing the profound influence of digital platforms in amplifying psychological vulnerabilities during and after exposure to armed conflict events.

### 1.5. The Developmental Perspective on Brain Changes from Adolescence to Adulthood

Recent studies have suggested that the transition from adolescence to early adulthood is marked by significant individual variation in structural brain development, making it a critical period for understanding ways in which social environments, personality traits, and affective states influence this development [53,61]. Adolescence is marked by engagement with complex mental states, emotions, and behaviors, although these emerging capacities remain unstable and can regress under stress or ambiguous conditions [56]. Mentalizing capacity, a crucial skill for social functioning and overall well-being, continues to develop during this period and does not fully mature until early adulthood. Neuroimaging studies further highlight significant differences in the activation of the mentalizing system between adolescents and young adults, particularly in tasks that require understanding oneself and others [62]. These developmental changes are associated with less efficient frontal activation in adolescents, limiting their ability to take perspectives compared to young adults [56]. Research suggests that the interplay between theory of mind and executive function improves significantly from adolescence into young adulthood, with notable advances observed in individuals aged 14–17 compared to those aged 19–27, underscoring the ongoing nature of these cognitive developments [63,64].

### 1.6. Adolescent Coping with Trauma

Adolescents exposed to online risks and traumatic experiences exhibit varying degrees of resilience, with some achieving positive psychological adjustment or experiencing a natural decline in symptoms over time [65,66,67,68]. Research has identified several personal factors that enhance adaptive coping following traumatic exposure. High levels of personal resources have been associated with lower maladjustment and fewer externalizing problems, while coping strategies such as problem-solving and seeking social support further contribute to resilience [69]. Additionally, religious coping has been linked to post-traumatic growth in adolescents, possibly due to the attribution of meaning [70]. Findings from studies on the COVID-19 pandemic showed that adolescents with higher baseline psychiatric symptoms experienced more severe distress post-lockdown. However, protective factors such as maintaining daily routines and receiving social support were found to buffer against these negative effects, promoting better mental health outcomes [71]. These findings indicate the importance of identifying potential resilience factors that may moderate adolescents’ ability to cope with trauma.

#### Self-Mastery as a Resilience Factor

Although the detrimental effects of internet and media exposure to armed conflict events have been well-documented, research has shown that post-exposure outcomes frequently range from severe pathology to unexpected resilience. This variability has led to the understanding that personal traits can play a significant role in shaping individual responses to such exposure [30,72]. Most existing research has focused on direct exposure to armed conflict events, identifying traits such as personal coping strategies [73,74], attribution of meaning [75,76], sense of coherence [77,78], and self-complexity [79] as moderating factors. Among the most commonly reported resilience factors in the context of traumatic exposure is self-mastery [80,81,82]. While these traits were shown to mitigate the adverse effects of direct trauma exposure, the present study examines the new question of whether self-mastery could serve as a potential resilience factor in the context of exposure to armed conflict events via the internet and social media. Further, the study examined this interaction of armed conflict media exposure and self-mastery across two age groups, namely adolescents and young adults.

Self-mastery pertains to self-belief in the ability to solve problems and take necessary actions to navigate the challenges of life [83]. The concept encompasses the perception of control in life, an internal sense of strength, and the capacity to cope with and overcome adversity [84]. Research has consistently linked high self-mastery to reduced psychological distress and fewer psychiatric symptoms [80,85,86], including a lower prevalence of post-traumatic symptoms [80,87].

The trait of self-mastery has been identified as a key moderating factor that mitigates the adverse effects of trauma and enhances recovery following various traumatic experiences [82,88,89]. In the context of armed conflict, self-mastery serves as a resilience factor [81,82] and may even contribute to post-traumatic growth [90]. Recent findings indicate that self-mastery not only has a direct protective effect against psychological symptoms but also mediates the relationship between exposure to life-threatening events and these symptoms [82]. However, exposure to severe trauma, such as armed conflict events, can sometimes result in a decline in self-mastery levels [80,91].

### 1.7. The Present Study

The present study aimed to investigate the psychological and psychiatric effects of internet media exposure to armed conflict events among two age groups, namely adolescents and young adults. Additionally, the study examined the potential moderating role of self-mastery as a resilience factor in the relationship between internet media exposure and psychological outcomes for each age group. This approach enables the identification of distinct characteristics of each group, offering a valuable developmental perspective. In order to isolate the effect of internet media exposure, direct exposure to armed conflict events was also included in the analysis.

### 1.8. Hypotheses

**Figure 1:**
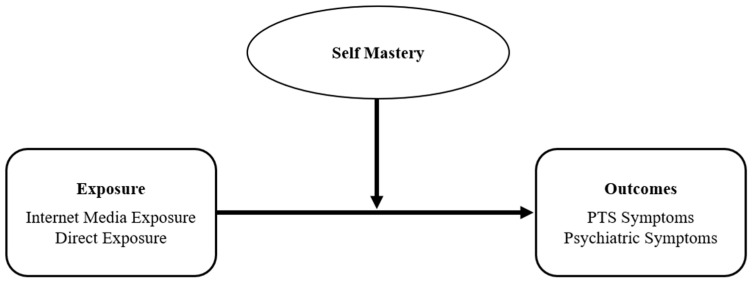
Conceptual diagram of the hypothesized relationships between the study variables.

The study posited three hypotheses for both age groups and an exploratory question addressing potential differences between the age groups, see Figure 1:

A. Direct Relationship between Internet Media Exposure and Symptoms: Hypothesis 1 posited a positive relationship between the extent of internet media exposure to armed conflict events and levels of post-traumatic symptoms and psychiatric symptomatology for each age group.
B. Direct Relationship between Direct Exposure and Symptoms: Hypothesis 2 posited a positive relationship between the severity of direct exposure to armed conflict events and levels of post-traumatic symptoms and psychiatric symptomatology for each age group.
C. Moderation Effect of Self-Mastery: Hypothesis 3 posited that self-mastery will moderate the relationship between exposure to armed conflict events, both direct and via the internet, and post-traumatic symptoms and psychiatric symptomatology for each age group.
D. Exploratory Question on Age Group Differences: An exploratory analysis aimed to investigate potential differences between adolescents and young adults in the effects of direct and internet-based exposure to armed conflict events, including the moderating role of self-mastery for each age group.

## 2. Materials and Methods

### 2.1. Participants

The study sample comprised 329 Israeli participants, including 159 adolescents aged 12 to 18 years (M = 15.81, SD = 1.58) and 168 young adults aged 20 to 26 years (M = 22.89, SD = 2.09). Demographic data indicated that the sample included participants from a range of socioeconomic backgrounds, spanning from low to high status. Among adolescents, 81.4% attended a regular school, 5.5% were enrolled in a special education school, and 13.1% were classified as “other”. Regarding young adults, 47.9% completed only high school education, 46.2% were either undergraduate students or held a BA degree, 3.6% were MA students or held an MA degree, and 2.4% were categorized as “other”. Participants were recruited via the internet, with informed consent obtained from both parents and adolescents for adolescent participants and from the participants themselves for young adults. Due to this recruitment method, gender was not evenly represented in the sample, with 69% female participants. Therefore, gender was included as a covariate in the statistical model to ensure that the effects were not influenced by the gender imbalance.

### 2.2. Instruments

#### 2.2.1. Direct Exposure to Armed Conflict Events

Direct exposure to armed conflict events was measured using a shortened version of the Political Life Events Scale (PLE; [92]), which assesses the severity of participants’ prior exposure to direct armed conflict events. Participants reported whether they had been exposed to 7 different events related to armed conflict, responding with either no exposure (0) or exposure (1). In the original version of the PLE, three levels of severity were included: 1 = low-severity events, 2 = moderate-severity events, and 3 = high-severity events. In the present study, only moderate and severe events from the PLE were included in the questionnaire, as mild events, such as attending a security drill or undergoing a security check at a mall entrance, are almost inevitably experienced routinely in Israel. For each event to which a participant was exposed, a value of 1 was assigned, which was subsequently multiplied by the corresponding weight: 2 for moderate severity events (e.g., “Time spent in a security shelter”), and 3 for high severity events (e.g., “Death of a family member as a result of an armed conflict event”). These weightings are the standard weightings assigned to the items in the PLE scale. The severity score was calculated by averaging weighted values assigned to each experienced event [79,93]. Therefore, the score ranged from 0 (representing no exposure to moderate or severe events) to 2.71 (representing the weighted average for exposure to all moderate and severe events). The PLE scale has been extensively used, and validity studies have demonstrated excellent results [93]. Internal consistency is not necessarily calculated for this scale as there is no theoretical rationale to expect consistency in exposure to discrete events [79,81]. High test–retest reliability was reported for the scale with scores ranging from r = 0.86 to r = 0.94 [79,94].

#### 2.2.2. Internet Media Exposure to Armed Conflict Events

Internet media exposure to armed conflict events was evaluated using a custom exposure scale designed for this study. Participants were asked to report the frequency with which they used various media platforms, including social media, internet news sites, and WhatsApp, after exposure to armed conflict events. Usage was rated on a scale from 1 (not at all) to 5 (all the time). A total internet media exposure score was calculated by averaging the ratings, ranging from 1 (indicating no exposure to any platform) to 5 (indicating exposure to all platforms at the most frequent level). Higher scores reflect greater exposure to media coverage of armed conflict events. Internal consistency was not calculated for this scale because there is no theoretical rationale to expect relations among the individual items.

#### 2.2.3. Self-Mastery

Self-mastery was assessed using the Mastery Scale [95], which measures overall perceived control. The scale consists of seven items, each rated on a seven-item point scale ranging from 1 (not at all) to 7 (very much). Example items include “What happens to me in the future mostly depends on me” and “I can do just about anything I really set my mind to do”. A total score was calculated by averaging responses to the items. Previous studies have reported test–retest reliability of 0.85 or higher and solid internal consistency (α = 0.75) [96]. In this study, the scale demonstrated an internal reliability of α = 0.80.

#### 2.2.4. Psychiatric Symptoms

Psychiatric symptoms were assessed using the Brief Symptom Inventory-18 (BSI-18) [97], a validated tool for screening psychological distress and psychiatric symptomology. The inventory includes 18 self-report items, with participants rating the frequency of symptoms experienced in the past month on a scale from 0 (not at all) to 4 (very much). Sample items include “feeling hopeless about the future”, “experiencing nervousness or inner shakiness”, and “feeling so restless that sitting still was impossible”. The BSI-18 provides scores for three subscales—depression, anxiety, and somatization—as well as a Global Severity Index (GSI), which is calculated as the mean of all item scores and serves as the primary measure of overall distress [97]. Previous studies have reported internal consistency values ranging from 0.74 to 0.89 [98]. In this study, the scale demonstrated strong reliability with a Cronbach’s alpha of α = 0.88.

#### 2.2.5. Post-Traumatic Symptoms

Post-traumatic symptoms were assessed using the PTSD Symptom Inventory [99], which includes 17 items aligned with PTSD criteria from the Diagnostic and Statistical Manual of Mental Disorders (DSM-IV; 4th ed.; [100]). Participants rated the combined frequency and severity of symptoms experienced over the past two weeks on a scale from 0 to 3 (0 = not at all, 1 = once per week or less, 2 = 2–4 times per week, 3 = 5 or more times per week). Examples of items include “having recurring nightmares or bad dreams about the trauma” and “feeling emotionally distant or disconnected from others since the trauma”. A total score was calculated by averaging the ratings, with higher scores indicating greater levels of post-traumatic symptoms. The inventory has demonstrated strong internal consistency (α = 0.85) and excellent interrater reliability for overall symptom severity (r = 0.97) [99], and it has been extensively used to study trauma related to terrorism, armed conflict, and other events [101]. In this study, the scale showed good internal consistency (α = 0.85).

### 2.3. Procedure

The study received approval from the Reichman University Ethics Committee and informed consent was obtained from all participants, with additional parental consent secured for adolescent participants. The questionnaire battery was then completed online via the Qualtrics survey platform, ensuring anonymity and confidentiality. Participants were informed that they could withdraw from the study at any time.

### 2.4. Data Analysis

In order to examine whether internet media exposure and direct exposure predicted psychiatric and post-traumatic symptoms as a function of self-mastery, structural equation modeling (SEM) was conducted using AMOS 29 with maximum likelihood estimation. A multi-group analysis was used to assess differences between adolescents and young adults in the various paths. This analysis involved fitting a general SEM model across all participants, as well as separate models for each group. Model comparisons were performed to determine which model, general or group-specific, provided a better fit to the data. When the separate models showed a better fit, specific paths were further tested by comparing a constrained model (with equal paths across groups) to an unconstrained model (with freely estimated paths). A significant difference between the models indicated a significant group difference in that path.

## 3. Results

### 3.1. Preliminary Analysis

Descriptive statistics, including means, standard deviations, and bivariate correlations for the study variables, are presented in Table 1 for adolescents (*n* = 159) and young adults (*n* = 168). Additionally, the table includes the results of independent sample t-tests conducted to assess group differences.

As presented in Table 1, adolescents reported lower levels of internet media exposure, direct exposure, and self-mastery compared to young adults. However, adolescents exhibited significantly higher levels of psychiatric and post-traumatic symptoms than young adults. Among adolescents, internet media exposure was positively associated with both psychiatric and post-traumatic symptoms, while no such association was observed among young adults. Self-mastery was negatively associated with both psychiatric and post-traumatic symptoms in both adolescents and young adults.

### 3.2. Mediation Moderation Analysis

In order to examine whether internet media exposure and direct exposure predicted both psychiatric and post-traumatic symptoms as a function of self-mastery, a structural equation modeling (SEM) approach was employed using AMOS 29 with maximum likelihood estimation. To assess differences between adolescents and young adults in the various paths, a multi-group analysis was used. Multi-group analysis in SEM is another form of moderation analysis using grouping variables. The model controlled for age and gender. Model fit was evaluated using four goodness-of-fit indices: the χ^2^ statistic, the Root Mean Square Error of Approximation (RMSEA), the Comparative Fit Index (CFI), and the Tucker–Lewis Index (TLI). A non-significant Chi-square indicates a good fit. Additionally, an RMSEA below 0.06 combined with CFI and TLI above 0.95 indicated excellent fit, while values below 0.08 and above 0.90, respectively, indicate adequate fit.

**Figure 2:**
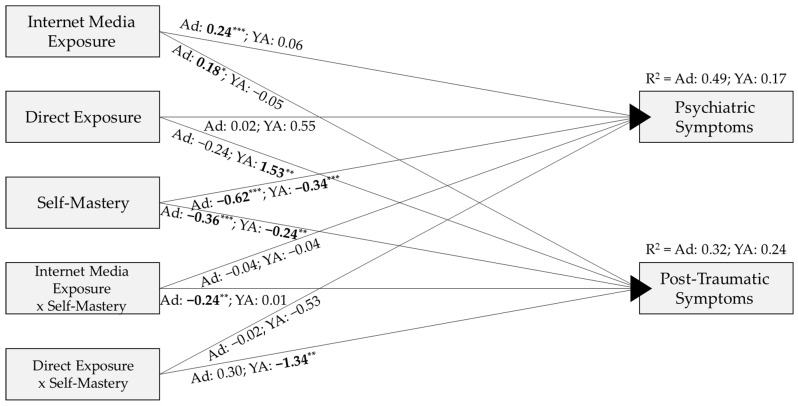
Structural equation model explaining the variance in psychiatric and post-traumatic symptoms by internet media exposure, direct exposure, and self-mastery. * *p* < 0.05; ** *p* < 0.01; *** *p* < 0.001. Notes. Ad = Adolescence; YA = Young Adults. Coefficients are standardized. Bold values are significant.

The results revealed a significant decrease in the goodness-of-fit indices for the constructed model (which forces the model paths to be equal across adolescents and young adults) compared to the unconstructed model (which allows separate paths for adolescents and young adults), χ^2^(14) = 34.06, *p* = 0.002. Specifically, these results suggest that the model with separate paths for adolescents and young adults fits the data better: χ^2^/df(22) = 1.56, *p* = 0.046, CFI = 0.99, TLI = 0.97, RMSEA = 0.042, in comparison to the model where adolescents and young adults are treated as a single group: χ^2^(36) = 1.90, *p* = 0.001, CFI = 0.98, TLI = 0.95, RMSEA = 0.053. Based on these findings, separate models are presented (see Figure 2). The covariance between the two dependent variables was included in the model as it was significant, with values of 0.72 in young adults and 0.48 in adolescents.

As illustrated in Figure 2, the first hypothesis, which predicted a positive relationship between the extent of internet media exposure to armed conflict events and the levels of post-traumatic symptoms and psychiatric symptomatology, was partially confirmed. This relationship was observed only among adolescents for both post-traumatic symptoms (β = 0.18, *p* < 0.05) and psychiatric symptomology (β = 0.24, *p* < 0.001). The relationship was not observed for young adults.

The second hypothesis posited a positive relationship between the severity of direct exposure to armed conflict events and levels of post-traumatic symptoms and psychiatric symptomatology. This hypothesis was partially confirmed, as the relationship was significant only for young adults and specifically only for post-traumatic symptoms (β = 1.53, *p* < 0.01).

**Figure 3:**
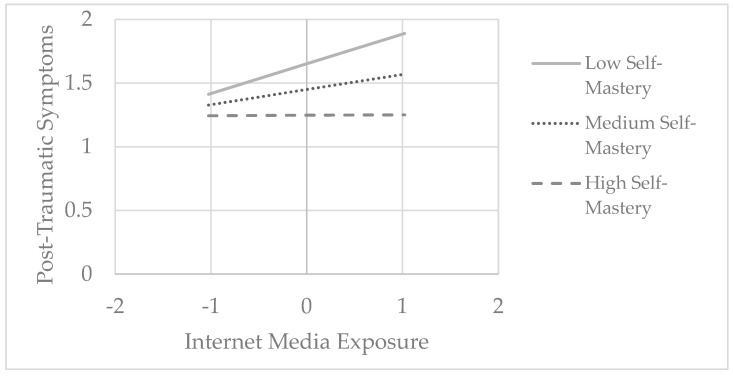
The relationship between internet media exposure and post-traumatic symptoms moderated by self-mastery among adolescents.

The third hypothesis predicted that self-mastery would moderate the relationship between exposure to armed conflict events, both direct and via the internet, and post-traumatic symptoms and psychiatric symptomatology. To examine significant interactions, Model 1 in PROCESS Macro V4.2 was used. This hypothesis was partially confirmed. Among adolescents, self-mastery significantly moderated the association only between internet media exposure and post-traumatic symptoms (F(1, 114) = 5.85, *p* = 0.017, ΔR^2^ = 0.036). A simple slope analysis revealed a significant positive effect of internet media exposure on post-traumatic symptoms among adolescents with low (−1 SD, Beta = 0.23, *p* < 0.001) and medium (Mean, Beta = 0.12, *p* = 0.013) levels of self-mastery. However, this effect was not observed among adolescents with high (+1 SD, Beta = 0.001, *p* = 0.960) levels of self-mastery (see Figure 3).

**Figure 4:**
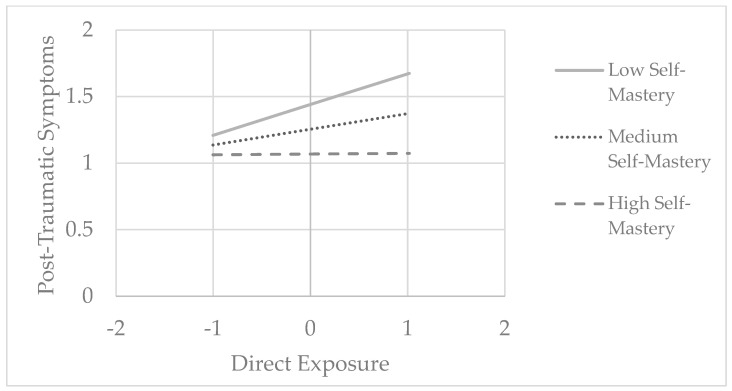
The relationship between internet media exposure and post-traumatic symptoms moderated by self-mastery among young adults.

Among young adults, self-mastery significantly moderated the association only between direct exposure and post-traumatic symptoms (F(1, 150) = 8.73, *p* = 0.004, ΔR^2^ = 0.044). A simple slope analysis revealed a significant positive effect of direct exposure on post-traumatic symptoms among young adults with low (−1 SD, Beta = 0.23, *p* = 0.002) and medium (Mean, Beta = 0.12, *p* = 0.011) levels of self-mastery. However, this effect was not observed among young adults with high (+1 SD, Beta = 0.01, *p* = 0.927) levels of self-mastery (see Figure 4).

The exploratory question related to possible age group differences in the effects of direct and internet media exposure to armed conflict events, including the moderating role of self-mastery for each age group. The answer to this question is reflected in the findings presented above, which highlight the different effects for each age group. For almost all paths that were significant in only one group, the difference between groups for the specific path was significant (*p* < 0.035). The only exception was the effect of internet media exposure on psychiatric symptoms, which was significant only among adolescents, however, the difference between groups for this path was not statistically significant.

## 4. Discussion

This study examined the effects of internet media exposure to armed conflict events on post-traumatic symptoms and psychiatric symptomatology among adolescents and young adults. Additionally, the moderating role of self-mastery as a resilience factor was explored. The results indicated that internet media exposure to armed conflict events was directly associated with psychiatric symptoms and post-traumatic symptoms only among adolescents. Conversely, prior direct exposure to armed conflict events was associated with post-traumatic symptoms only among young adults. The moderating effect of self-mastery was confirmed for both age groups, as self-mastery moderated the effect of the type of exposure relevant to each group. Specifically, for adolescents, self-mastery moderated the relationship between internet media exposure and post-traumatic symptoms, whereas for young adults, self-mastery moderated the relationship between direct exposure and post-traumatic symptoms. These results also shed light on the exploratory question regarding the differences between the two age groups, which is discussed in relation to each finding.

The first hypothesis, which predicted a positive relationship between the extent of internet media exposure to armed conflict events and levels of post-traumatic symptoms and psychiatric symptomatology, was confirmed only for adolescents and not for young adults. This finding aligns with previous research highlighting the role of the media as a powerful tool through which armed conflict events and terrorism instill fear and a sense of threat. These negative effects reach communities and individuals distant from the actual site of the attack, with detrimental effects among adolescents [19,24,25]. One possible explanation for this outcome is that adolescents may lack the cognitive maturity required to critically evaluate the authenticity of media content, making them less capable of discerning fact from fiction. Furthermore, adolescents often lack the cognitive and emotional resources needed to distance themselves from the media content and engage in rationalization. Due to the developmental characteristics of this life stage, which is marked by a search for identity and a heightened susceptibility to external influences [40,41], adolescents may be particularly vulnerable to the psychological impacts of violent media content. Moreover, adolescents tend to experience greater fluctuations in mood and affect than young adults. As a result, they may have a lower threshold for perceiving an event as traumatic, making them more susceptible to distress from media exposure to armed conflict [56]. Additionally, adolescents are often highly proficient in using new media platforms, many of which are uncensored and unsupervised, exposing them to graphic and often disturbing footage that they may struggle to process. Given their developmental stage, adolescents may find it particularly challenging to regulate their emotional responses to such distressing material.

Unlike adolescents, who may lack the ability to critically assess media content, young adults may have a heightened awareness of the potential harm associated with exposure to graphic or horrifying scenes and are better able to distinguish between true and false information [31]. This enhanced ability to critically evaluate media is further supported by young adults’ greater understanding of the motivations behind media content, such as its tendency to dramatize or evoke emotional responses. In contrast to adolescents, young adults are likely to be more cautious in their media consumption. This difference can be attributed to their greater cognitive maturity and more developed coping mechanisms [51,52]. Moreover, the impact of media tends to be more pronounced on adolescents due to their less stable cognitive and emotional regulation. As Wallace suggests in [31], the level of trust and ability to think critically about media may influence whether the news is perceived as conveying a realistic threat. This difference is rooted in developmental factors. Young adults possess more mature mentalizing capacities, allowing them to engage with complex mental states, emotions, and behaviors in a more stable manner compared to adolescents, whose mentalizing abilities are relatively unstable and can regress in ambiguous or threatening conditions [56]. Thus, young adults may be better equipped to navigate the complexities of media content and its potential psychological impacts.

The second hypothesis, which predicted a positive relationship between the severity of direct exposure to armed conflict events and levels of post-traumatic symptoms and psychiatric symptomatology, was confirmed only among young adults and specifically for post-traumatic symptoms. The emergence of post-traumatic symptoms following exposure to such traumatic events is expected and well-documented in the literature [25,72,79,102]. Moreover, this finding emphasizes the severity of the experience, particularly given that the measure used in this study included only moderate and severe items related to armed conflict and excluded mild events. The lack of effect among adolescents may be attributed to the accumulation of exposure to armed conflict events over time. Young adults are likely to have a history of more frequent and severe exposures compared to adolescents who have had fewer years of potential exposure and may still be partially shielded by parental protection [50,51].

The third hypothesis concerned the possible moderating effect of self-mastery, positing that self-mastery would moderate the relationship between exposure to armed conflict events, both direct and via the internet, and the post-traumatic outcome measures. This hypothesis was confirmed for both age groups with regard to post-traumatic symptoms but for different types of exposure. Among adolescents, self-mastery moderated the effects of internet media exposure on post-traumatic symptoms, such that adolescents with high self-mastery exhibited lower levels of post-traumatic stress symptoms compared to those with low self-mastery. Among young adults, a similar pattern emerged but for the direct exposure variable, whereby self-mastery moderated the effects of direct exposure on post-traumatic symptoms. Young adults high in self-mastery showed lower levels of post-traumatic stress symptoms compared to those with low self-mastery.

This finding offers a hopeful contrast to the typically negative outcomes predicted in the field of trauma research as it supports the idea that personal traits such as self-mastery can mitigate the severe effects of traumatic exposure [72]. While the moderating role of self-mastery has been well-documented [81,82], this study is a pioneer in showing its moderating effect for both direct and indirect internet media exposure across two distinct age groups. Notably, for each age group, self-mastery moderated the type of exposure relevant to each group. For adolescents, exposure to horrific graphic scenes in internet media predicted higher levels of post-traumatic symptoms, with self-mastery serving as a protective factor. For young adults, severe direct exposure to armed conflict events was associated with post-traumatic symptoms, which was moderated by the capacity for self-mastery. These findings suggest that the most relevant form of trauma is the one that evokes anxiety and post-traumatic symptoms, requiring individuals to adjust to difficult circumstances. In this context, self-mastery, which represents self-belief in the ability to solve problems and take necessary actions to navigate challenges, acts as a resilience factor, protecting individuals from the overwhelming effects of trauma that could otherwise induce feelings of helplessness. Thus, these results highlight the critical role of self-mastery in coping with and adjusting to traumatic exposures.

The finding that self-mastery moderated the effects of internet media exposure on post-traumatic symptoms among adolescents highlights the unique vulnerability of this age group to online content and sheds light on a potential way to mitigate the impact of this problem. This finding suggests that while adolescents are increasingly exposed to global events through digital platforms, their ability to cope with such exposure is influenced by their self-mastery. Since adolescents have less ability to navigate media effectively, they are more likely than adults to experience heightened emotional reactions to media content [56]. However, the findings here show that adolescents with high self-mastery are better equipped to manage the emotional intensity and stress induced by exposure to violent or traumatic media, emphasizing the importance of fostering this trait during adolescence. Moreover, the findings of this study suggest that developing clinical interventions aimed at enhancing self-mastery among individuals, particularly adolescents, is crucial and could contribute to better mental health outcomes.

### Study Limitations

Despite these innovative findings and their theoretical and practical implications, this study has several limitations that should be addressed in future research. First, the research design relied on single-agent, self-reported questionnaires administered online. While this method allowed for the assessment of uncensored and distressing media exposure, which is an essential aspect given its widespread availability on the internet, self-report also introduces potential response biases and limits the ability to establish causality. Ethical considerations prevent the deliberate exposure of participants to such content in a laboratory setting due to the potential emotional harm, necessitating a field study approach. To minimize potential biases, efforts were made to ensure complete anonymity and confidentiality, utilizing validated scales, and encouraging honest responses by emphasizing that there are no right or wrong answers. Future studies could mitigate these limitations by incorporating multi-agent reports or behavioral measures to enhance data reliability and validity.

Second, this study focused solely on self-mastery as a resilience factor. While self-mastery proved to be a significant protective mechanism, other resilience factors such as social support, adaptive coping strategies, and the attribution of meaning to traumatic experiences may also play a crucial role in buffering the negative psychological effects of both internet media exposure and direct exposure to armed conflict. Future research should explore these additional resilience mechanisms to provide a more comprehensive understanding of the factors that promote psychological well-being in the face of traumatic exposure. Third, the potential for cohort effects should be acknowledged. Since this study relied on cross-sectional data, it is possible that the differences between adolescents and young adults were related to generational differences, such as historical events beyond the immediate context of armed conflict. These events, including economic recessions, pandemics, or cultural shifts, may have shaped the beliefs, behaviors, and vulnerabilities of different age groups.

Finally, this study was conducted within the context of an ongoing armed conflict, which limits the generalizability of the findings to other types of trauma characterized by isolated, single-event exposures. Additionally, the applicability of these results to other forms of trauma, such as sexual assault or natural disasters, may be constrained. Future research could benefit from examining the moderating role of self-mastery in these different traumatic contexts to better understand its broader relevance.

## 5. Conclusions

This study provides important insights into the role of self-mastery in moderating the psychological impact of exposure to armed conflict events, both directly and through internet media, among adolescents and young adults. The findings reveal that while adolescents are particularly vulnerable to the psychological effects of internet media exposure, young adults experience post-traumatic symptoms primarily in response to direct exposure. The findings highlight the heightened vulnerability of adolescents to online traumatic content, reinforcing the urgent need for protective measures. However, in both age groups, self-mastery emerged as a crucial resilience factor, buffering the adverse psychological effects of trauma exposure. These results underscore the importance of fostering self-mastery as a protective mechanism, particularly for adolescents who frequently engage with unfiltered and graphic content online.

The findings of this study offer several important implications for public policy, education, and clinical interventions. For adolescents, policymakers and educational institutions should prioritize programs aimed at strengthening a sense of agency and self-mastery, equipping adolescents with essential skills to regulate emotions, process traumatic content, and navigate media critically. Public health initiatives should also focus on increasing parental awareness regarding the psychological risks associated with unsupervised exposure to violent media while providing guidance on resilience-promoting parenting strategies. Due to the difficulty of monitoring adolescent media consumption, given their developmental need for autonomy, educational campaigns targeting parents and caregivers are crucial. For young adults, while media exposure remains a concern, direct exposure to armed conflict poses an even greater psychological risk. As such, intervention efforts should extend beyond media literacy programs to include primary and secondary prevention strategies that address trauma-related distress and promote adaptive coping mechanisms. The findings on young adults are not only significant for this age group but also offer valuable insights regarding the future risks to adolescents transitioning into adulthood in armed conflict areas. Examining young adults enabled a deeper understanding of the expected developmental trajectory of adolescents exposed to such traumatic events, providing a broader perspective on how traumatic exposure, resilience, and coping strategies evolve over time.

Mental health practitioners working with individuals from conflict-affected regions should integrate resilience-based therapeutic approaches that enhance self-mastery, enabling individuals to regain a sense of control and psychological stability. Beyond individual interventions, the broader societal impact of armed conflict on adolescents and young adults must be acknowledged. War and armed conflict disrupt not only individual mental health but also the social structures that support development, including families, schools, and communities. Clinical interventions should, therefore, adopt a multi-layered approach, targeting both individual resilience factors and the reinforcement of familial and societal support networks. Additionally, this study highlights the ethical and regulatory challenges posed by the widespread dissemination of violent content on the internet and social media. Public policy should encourage the development of guidelines for internet and social media platforms to monitor and limit exposure to particularly distressing content. Media literacy initiatives should be integrated into school curricula, equipping adolescents with the critical thinking skills needed to assess and interpret violent content in a more constructive manner.

Given the increasing prevalence of digital media as a primary source of information, future research should explore the long-term effects of media exposure to traumatic events and examine additional protective factors that may further support resilience in young people. The findings of this study highlight the need for developmentally sensitive community programs that address the psychological challenges faced by youth in a cost-effective and accessible manner. By integrating resilience-building initiatives and by addressing the ethical complexities of media exposure, policymakers, educators, and mental health professionals can work collaboratively to safeguard the well-being of adolescents and young adults in an era of pervasive direct and digital trauma exposure.

## Figures and Tables

**Table 1:** Means, standard deviations, and bivariate correlations of study variables for adolescents (*n* = 159) and young adults (*n* = 168).

	1	2	3	4	5
Internet Media Exposure	-	0.11	0.18 *	0.00	−0.08
2. Direct Exposure	−0.12	-	−0.07	0.02	0.17 *
3. Self-Mastery	−0.07	0.04	-	−0.33 ***	−0.32 ***
4. Psychiatric Symptoms	0.32 ***	−0.06	−0.65 ***	-	0.74 ***
5. Post-Traumatic Symptoms	0.26 **	0.05	−0.45 ***	0.65 ***	-
Adolescents					
M	2.36	0.43	3.64	2.33	1.44
SD	0.66	0.37	0.81	0.86	0.61
Young Adults					
M	2.64	0.64	3.87	1.80	1.29
SD	0.60	0.50	0.53	0.82	0.65
t-test	−3.81 ***	−4.31 ***	−2.98 **	5.71 ***	2.18 *

* *p <* 0.05; ** *p* < 0.01; *** *p* < 0.001; Note. The correlations above the diagonal were calculated for young adults (*n* = 168), while those below the diagonal were calculated for adolescents (*n* = 159).

## Data Availability

The raw data supporting the conclusions of this article will be made available by the authors on request.
